# Improving adherence to medication in adults with diabetes in the United Arab Emirates

**DOI:** 10.1186/s12889-016-3492-0

**Published:** 2016-08-24

**Authors:** Mohammed M. M. Al-Haj Mohd, Hai Phung, Jing Sun, Donald E. Morisky

**Affiliations:** 1 School of Public Health, Griffith University, Gold Coast, Australia; 2 Dubai Police Health Centre, Dubai, United Arab Emirates; 3 Department of Community Health Sciences, UCLA Fielding School of Public Health, Los Angeles, U.S.A

**Keywords:** Diabetes, Adherence, Medication

## Abstract

**Background:**

Diabetes is a chronic medical condition and adherence to medication in diabetes is important. Improving medication adherence in adults with diabetes would help prevent the chronic complications associated with diabetes. A case control trial was used to study the effects of an educational session on medication adherence among adults with diabetes as measured by the Morisky Medication adherence scale (MMAS-8©).

**Methods:**

The study took place at the Dubai Police Health Centre between February 2015 and November 2015. Questionnaires were used to collect socio-demographic, clinical and disease related variables and the primary measure of outcome was adherence levels as measured by the Morisky Medication Adherence Scale (MMAS-8©). The intervention group involved a standardized thirty minute educational session focusing on the importance of adherence to medication. The change in MMAS-8© was measured at 6 months.

**Results:**

Four hundred and forty six patients were enrolled. Mean age 61 year +/− 11. 48.4 % were male. The mean time since diagnosis of diabetes was 3.2 years (Range 1–15 years). At baseline two hundred and eighty eight (64.6 %) patients were considered non-adherent (MMAS-8© adherence score < 6) while 118 (26.5 %) and 40 (9.0 %) had low adherence (MMAS-8© adherence score < 6) and medium adherence (MMAS-8© adherence scores of 6 to 7) to their medication respectively. The percentage of patients scoring low adherence MMAS-8 scores in the interventional group dropped from 64.60 % at baseline to 44.80 % at 6-months (*p* = 0.01). There was no obvious change in the adherence scores at baseline and at 6-months in the control group. Based on the study data, the Wilcoxon signed-rank test showed that at 6 months, the educational 30-min session on diabetes and adherence to medication did elicit a statistically significant change in adherence levels in adults with diabetes enrolled in the intervention arm (*Z* = −6.187, *p* <0.001).

**Conclusion:**

Adults with diabetes would benefit from educational sessions focusing on the importance of adherence to medication. Public health strategies should focus on wider educational strategies targeting medication adherence in diabetic patients in the UAE.

## Background

Diabetes mellitus is a chronic metabolic disease affecting approximately 341 million to 371 million people worldwide [1, 2]. Furthermore, it is estimated that one third of those affected (approximately 122.5 million) are not aware that they have the condition [3].

The oil boom has led to a massive increase in the GDP and disposable income of the people of the United Arab Emirates (UAE). The UAE was ranked as the 19th highest income countries of the world in 2012 (International Monetary Fund) and is categorized as a high income country according to the (World bank, 2012). This has led to a more affluent lifestyle, and from health point an increase in total calorie intake per person together with a decrease in calorie expenditure. This has led to a nationwide obesity pandemic with the rates of obesity climbing to record highs and standing at about 68 % of the population according to one estimate from 2007 .

The UAEis now listed as the country with the 11th highest prevalence of diabetes globally (primarily type-2) [4]. Furthermore, metabolic control of diabetes is reportedly poor, leading to an increased risk of associated complications [5]. Almost 70 % of Emirati nationals are reported to be overweight or obese [6], and one third of Emirati children are also now obese [7]; these figures are two to three times those of international standards [8], thus, it is unsurprising that the prevalence of type-2 diabetes has escalated.

Medication non-adherence is of increasing concern for healthcare providers despite the known benefits of modern treatment regimens, with prevalence reported in one study to be in excess of 50 % of diabetic patients. The consequences of non-adherence include not only health-related consequences (i.e. failure of treatment, rehospitalisation, death), but also financial consequences as the cost of emergency medical interventions as a consequence of non-adherence outweigh the combined cost of an adhered-to medication regimen [9]. The WHO has identified non-adherence as a multifactorial problem caused by the interplay of factors from any of the following 5 areas: 1) the patient, 2) the condition, 3) the type of therapy prescribed, 4) socioeconomic factors, and 5) health system related factors [10]. Several studies have been carried out looking at medication adherence in diabetic patients around the world; however no studies have been performed in the U.A.E [11–13].

## Methods

### Study design

A case control trial was used to study the effects of an educational session on medication adherence among adults with diabetes as measured by the Morisky Medication adherence scale (MMAS-8©). Patients were randomized to standard care (control group) or standard care plus an educational session (intervention group) on the importance of adherence to medication. The MMAS-8 © scores were collected via questionnaires at baseline and at the end of the study. The change in MMAS-8 © scores was analysed using statistical software. The research topic was granted an ethical clearance through the Human Research Ethics Committee at Griffith University GU Ref No: PBH/11/14/HREC and confirmed with the Dubai Police Research Ethics standards.

### Study setting

The study took place at the Dubai Police Health Services Clinic between February 2015 and November 2015. This centre provides primary care and speciality care for all Dubai Police employees and members of their families and had over 200,000 clinic visits in 2014 alone.

### Procedures (Fig. 1)

**Fig. 1:**
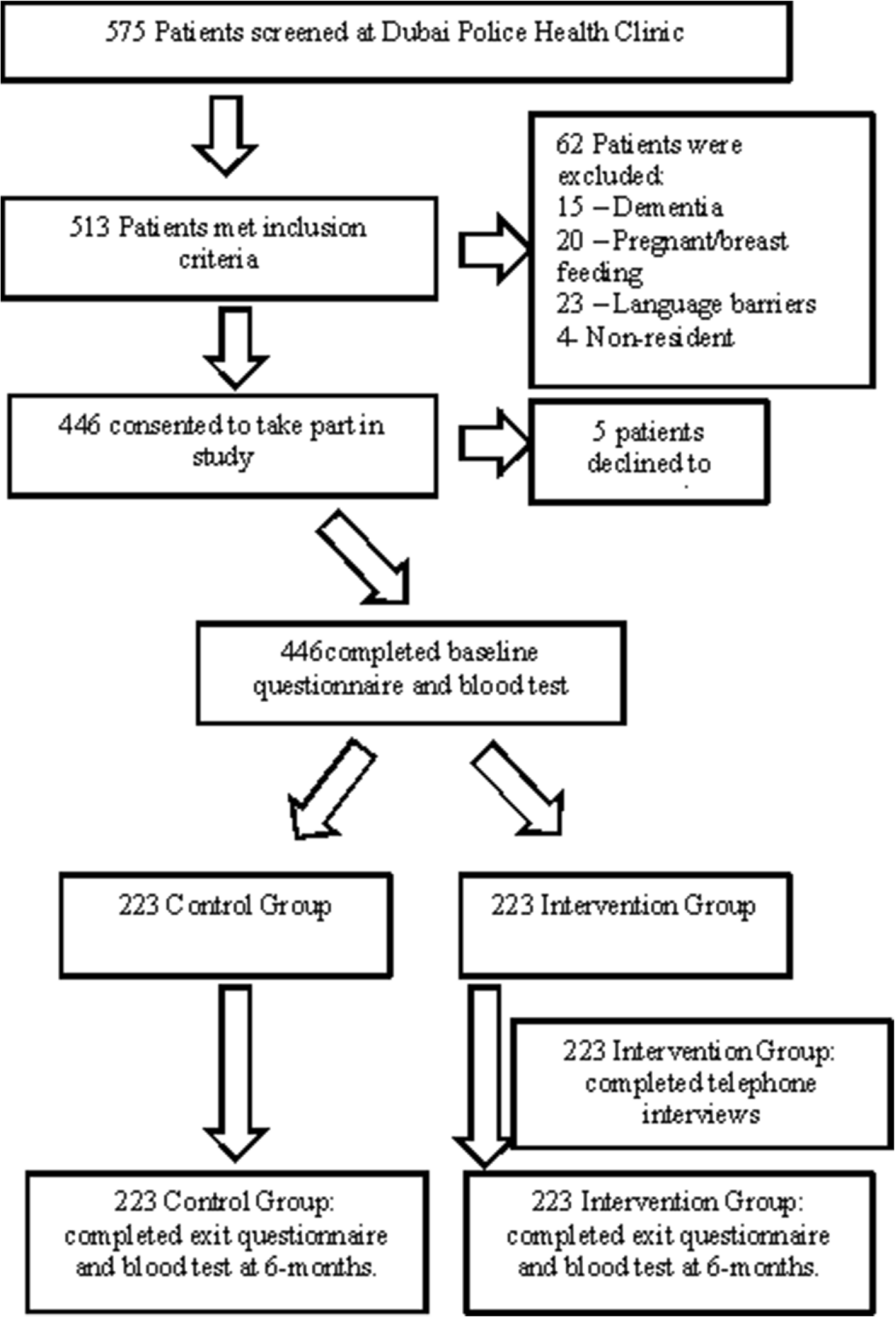
Recruitment and randomization process

#### Inclusion criteria

All patients were type 2 adults with diabetes on at least one anti-diabetic medication following a diabetes diagnosis of 1 year or more. Male and female patients between the age of 18 and 80 years were eligible for the study. The following exclusions applied: cognitive impairment, pregnant or breast feeding women, non-residents and patients who did not comprehend either the English or Arabic language.

The investigators identified and screened potential participants in the following manner: all patients present in the waiting area of the diabetic clinic were asked if they were willing to talk to the investigator. If the person agreed, then an informed consent was read and explained by the investigators. Once verbal consent was obtained, the inclusion and exclusion criteria were checked.

Data was collected primarily via questionnaires. Patients were asked to fill in these questionnaires in order to gather demographic information and information regarding motivations and associated behaviours. These questionnaires were written using the Android Database software package (MEMENTO). The questionnaires were then handed out in electronic form using an Android tablet. The tablets were purchased for the purpose of this research and were solely used for this purpose. These tablets were secured with an encrypted password and were locked in drawer at the research office when not in use. The tablets and questionnaires were supervised by one of the research investigators at the time of interview to ensure the correct handling of the questionnaire and to troubleshoot any problems if they would arise. The questionnaire forms were based on a number of field styles including free text, date, single best answer and multi-check selections. The database software collated the results into an excel sheet which was later exported to a computer for further data analysis. All participants completed the questionnaires in a private clinic room.

A simple random number generator was used to randomly allocate patients to either the control group or the intervention group. The intervention group then went on to attend a 30-min education session about diabetes and its associated medication (this group is referred to as the ‘intervention group’), whilst the other group was not invited to attend this session, and instead received the same level of education regarding diabetes as is offered as standard from UAE healthcare providers (this group is referred to as the ‘control group’). Following which, the intervention group received a weekly phone call to check on their progress for the duration of 3 months, the control group were not eligible for these phone calls. The follow-up phone call served more of a motivational strategy by asking the patients standardized questions like: 1- Do you have any concerns about your diabetes medication? – Interviewer to try and answer the patients concerns and refers to clinician as appropriate. 2- Did you face circumstances that have prevented you from picking up your medication from your pharmacy? Interviewer: To try and address these concerns and refer to social worker as appropriate.

The patients were then re-invited to undertake the questionnaires at 6 months from the time of their initial visit in order to determine any long-term improvements in medication adherence. Furthermore, whole blood samples were collected by venepuncture from all patients at their initial and 6-month visits in order to determine HbA1c concentrations.

### Educational session on adherence to medication

Patients randomized to the intervention arm were subjected to a 30 min educational session focusing on the importance of medication adherence. The educational session was undertaken by a diabetes nurse and consisted of a 30 min session aided with a PowerPoint presentation based on slides adopted from the International Diabetes Federation (IDF) education modules published online in 2011. The PowerPoint presentation was structured around medication adherence and compliance. The importance of medication adherence as well as barriers to self-management were covered and explained by covering the following themes:

Check that the patient understands when and how they should take their medicine.

Ask the patient when they will take the medicine.

Explain the benefits of the medicine—stress the fact that patients may not have any symptoms of diabetes or feel at all ill; therefore unlikely that the medicine will make them “feel better”.

Be sure it is understood that the medicine is having a beneficial effect inside the body.

Ensure that people know what to expect in terms of side effects, and that these might only be short term; thus people will be more likely to continue with the medicine (another reason people stop is that they did not like the side effects).

The aim of the interview was to educate the patients on the importance of medication and the importance of adherence in the long term on the outcomes of their diabetes. Breifly, the investigators would have explained the action of each diabetes medication, the time at which the medication was to be taken and the benefits of taking these medications. These points would have re—inforced the patients’ knowledge on diabetes management and medication compliance.

The didactive nature of the slide show presentation was coupled with a more collaborative approach where the diabetes educator would encourage the patient to ask questions about medication adherence and answer those questions.

### Data variables

The data collated via the questionnaires included the following variables: age, gender, ethnicity, marital status, highest level of education attained, working conditions, transport availability, smoking status, diabetes duration, cultural factors (dress wear, behaviors in Ramadan, perception towards obesity), number of anti-diabetic medication, insulin therapy, the Depression, Anxiety and Stress scale (DASS-21) and International Physical Activity Questionnaire (IPAQ) score. The Morisky Medication Adherence Scale −8 (MMAS-8) score was used to measure the outcome of adherence to medication. MMAS-8. The Glycated Haemoglobin test (HbA1c (%)) was used to check the validity of the MMAS-8 score in this cohort as a measure of adherence to medication among the study participants. IBM SPSS-20 was used for statistical analyses.

#### Morisky medication adherence scale

The Morisky Medication Adherence Scale (MMAS) was designed to determine adherence behaviours [14]. For this study the 8-item model of this scale was used; patients were asked 8 questions (Table 1) designed to determine which factors affect how well they adhered to their medication regimen. Patients are required to answer the questions with either a ‘yes’ or a ‘no’, with the final question taking the form of a typical five-point Likert item. Positive answers (i.e. yes) are scored a 1 and negative answers (i.e. no) are scored a 0. From these responses a final score was calculated with three possible outcomes; a score of >2 corresponded to low medication adherence, a score of 1 or 2 corresponded to medium medical adherence, and a score of 0 corresponded to high medical adherence. The MMAS is a popular, easy and economical method of data collection, facilitating the collection of a large amount of data in a short period of time [15].

**Table 1:** The 8 questions asked to determine medication adherence based on the morisky medication adherence scale

Questions
1. Do you sometimes forget to take your medicine?
2. People sometimes miss taking their medicines for reasons other than forgetting. Thinking over the past 2 weeks, were there any days when you did not take your medicine?
3. Have you ever cut back or stopped taking your medicine without telling your doctor because you felt worse when you took it?
4. When you travel or leave home, do you sometimes forget to bring along your medicine?
5. Did you take all your medicines yesterday?
6. When you feel like your symptoms are under control, do you sometimes stop taking your medicines?
7. Do you ever feel hassled about sticking to your treatment plan?
8. How often do you have difficulty remembering to take all your medicine?
1. Never
2. Once in a while
3. Sometimes
4. Usually
5. All the time

## Results

A total of 513 patients were identified as meeting the study inclusion criteria, and 442 patients agreed to take part of the study. There were no patients lost to follow-up (Fig. 1). There were 223 patients in the control group and 223 patients in the control group. The baseline characteristics were well distributed between the two groups with no significant differences among the two groups. 48.4 % (*n* = 216) of patients were male. The mean time since diagnosis of diabetes was 3.2 years (Range 1–15 years). Emaratis represented 56.1 % of the study population patients, followed by Arab Non Emarati patients (38.1 %) and Asian patients (5.8 %) (Tables 2 and 3).

**Table 2:** Baseline characteristics

		Randomization Group	
		Control	Intervention
Age (mean +/− std)		61_a_	62_a_
		+/− 11	+/− 11
Gender	Female	52.0 %_a_	51.1%_a_
		116	114
	Male	48.0 %_a_	48.9%_a_
		107	109
Ethnicity	Arab Emarati	53.8%_a_	58.3%_a_
		120	130
	Arab Non-Emarati	40.8%_a_	35.4 %_a_
		91	79
	Asian	5.4 %_a_	6.3 %_a_
		12	14
HbA1c baseline (mean +/− std)		8.50_a_	8.49_a_
		.09	.10
SBP at baseline (mean +/− std)		134_a_	133_a_
		26	26
DBP at baseline (mean +/− std)		72_a_	73_a_
		20	21
HDL at baseline (mean +/− std)		54_a_	54_a_
		11	11
LDL at baseline (mean +/− std)		130_a_	129_a_
		36	37
TGL at baseline (mean +/− std)		207_a_	216_b_
		42	41
Anti-Diabetic therapy	Monotherapy	27.8%_a_	30.9%_a_
		62	69
	Combination	72.2%_a_	69.1%_a_
		161	154
Insulin use	Yes	49.8%_a_	50.7 %_a_
		111	113
	No	50.2%_a_	49.3%_a_
		112	110
Prescence of Chronic conditions	Yes	52.9%_a_	55.2%_a_
		118	123
	No	47.1%_a_	44.8%_a_
		105	100

Note: Values in the same row and subtable not sharing the same subscript are significantly different at *p* < .05 in the two-sided test of equality for column proportions. Cells with no subscript are not included in the test. Tests assume equal variances.^1^

**Table 3:** Adherence levels against different demographic variables

		Adherence level								
		Low Adherence (MMAS-8 < 6)			Medium Adherence (MMAS-8 = 6 to 7)			High Adherence (MMAS-8 = 8)		
		Row %	Column %	n	Row %	Column %	n	Row %	Column %	n
Marital Status	Single	70.4 %	6.6%_a_	19	18.5 %	4.2%_a_	5	11.1 %	7.5%_a_	3
	Married	66.0 %	88.2%_a_	254	26.5 %	86.4%_a,b_	102	7.5 %	72.5%_b_	29
	Divorced	51.9 %	4.9%_a_	14	37.0 %	8.5%_a_	10	11.1 %	7.5%_a_	3
	Widowed	14.3 %	0.3 %_a_	1	14.3 %	0.8%_a_	1	71.4 %	12.5%_b_	5
Living arrangements	Alone	79.0 %	17.0 %_a_	49	17.7 %	9.3%_a_	11	3.2 %	5.0 %_a_	2
	Partner with children	63.9 %	75.7 %_a_	218	26.7 %	77.1%_a_	91	9.4 %	80.0 %_a_	32
	Partner with no children	42.9 %	2.1%_a_	6	28.6 %	3.4%_a,b_	4	28.6 %	10.0 %_b_	4
	Single adult with children	50.0 %	3.5%_a_	10	45.0 %	7.6 %_a_	9	5.0 %	2.5%_a_	1
	Other family members	55.6 %	1.7%_a_	5	33.3 %	2.5%_a_	3	11.1 %	2.5%_a_	1
Level of Education	Primary/Secondary school	76.8 %	36.8%_a_	106	21.0 %	24.6%_a,b_	29	2.2 %	7.5%_b_	3
	High school school	79.7 %	34.0%_a_	98	17.9 %	18.6%_b_	22	2.4 %	7.5%_b_	3
	Technical diploma	43.3 %	13.5%_a_	39	46.7 %	35.6%_b_	42	10.0 %	22.5%_a,b_	9
	University degree	47.4 %	15.6%_a_	45	26.3 %	21.2%_a_	25	26.3 %	62.5%_b_	25
Working conditions	Employed	63.6 %	85.4 %_a_	246	27.4 %	89.8%_a_	106	9.0 %	87.5%_a_	35
	Unemployed	81.1 %	10.4%_a_	30	18.9 %	5.9%_a_	7	0.0 %	0.0 %^1^	0
	Retired	42.9 %	2.1%_a_	6	21.4 %	2.5%_a_	3	35.7 %	12.5%_b_	5
	Sickness beneficiary	75.0 %	2.1%_a_	6	25.0 %	1.7%_a_	2	0.0 %	0.0 %^1^	0
Transportation Avaliability	No	70.4 %	6.6 %_a_	19	29.6 %	6.8%_a_	8	0.0 %	0.0 %^1^	0
	Yes	64.2 %	93.4%_a_	269	26.3 %	93.2%_a_	110	9.5 %	100.0 %^1^	40

Note: Values in the same row and subtable not sharing the same subscript are significantly different at *p* < .05 in the two-sided test of equality for column proportions. Cells with no subscript are not included in the test. Tests assume equal variances.^2^

1. This category is not used in comparisons because its column proportion is equal to zero or one

2. Tests are adjusted for all pairwise comparisons within a row of each innermost subtable using the Bonferroni correction

**Fig. 2:**
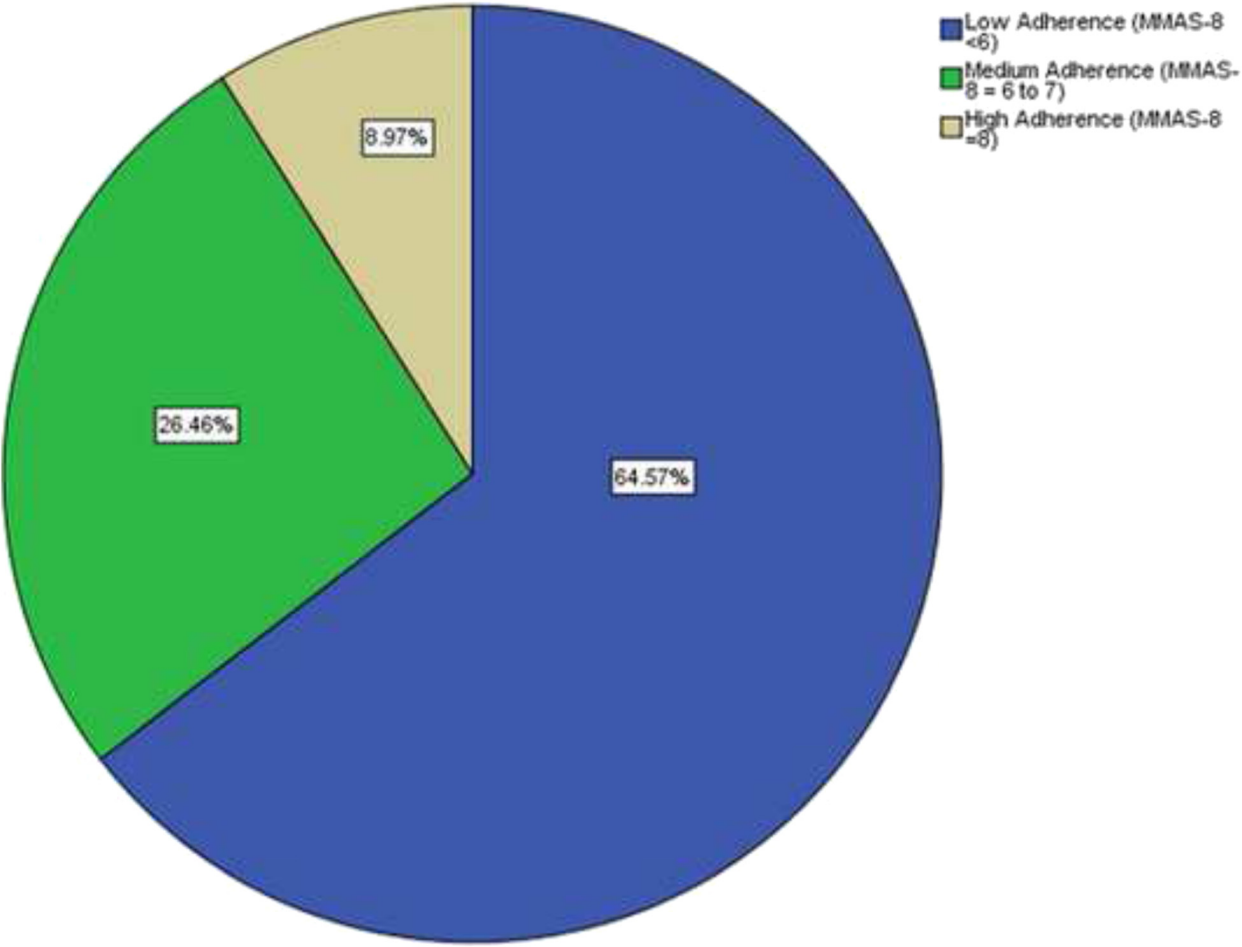
Rates of adherence according to MMAS-8 scores

Two hundred and eighty eight (64.6 %) patients were considered non-adherent (MMAS-8© adherence score <6) while 118 (26.5 %) and 40 (9.0 %) had medium adherence (MMAS-8© adherence score 6 to 7) and high adherence (MMAS-8© adherence scores of <8) to their medication respectively (Fig. 2).

**Fig. 3:**
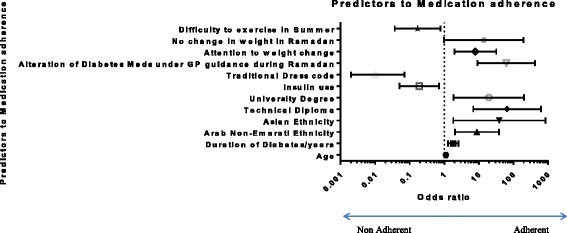
Predictors to medication adherence

The strongest predictor for adherence as predicted by the multi-logistic regression model was the patient’s level of education. A technical diploma certificate as compared to a primary school level of education was the strongest predictor of adherence (*OR* = 66.1 CI: 6.93 to 630.43); *p* < 0.001). The patient’s age was also a predictor of adherence with older patients reporting higher levels of adherence (*OR* = 1.113 (CI: 1.045 to 1.185; *p* = 0.001 for every year increase in age). The duration of diabetes was also a predictor of adherence (*OR* = 1.830 (CI: 1.270 to 2.636; *p* = 0.001 for every year increase in the duration of diabetes). Other predictors to medication adherence include Insulin use, ethnicity and certain cultural behaviours (Fig. 3).

### Reliability, internal consistency and validity of MMAS-8

The Cronbach’s alpha test was calculated for the 8-item MMAS-8 and this was reliable at 0.736 Omission of any of the 8-items of the MMAS-8 questionnaire resulted in a lower Cronbach’s alpha. The validity of MMAS-8 adherence score was assessed by testing the ability of the score to distinguish between groups of individuals that differ from each other according to the HbA1c. There was a significant difference in the Mean HbA1c levels among the three adherence groups. Mean HbA1c was 9.24, 7.33 and 6.60 % in the low, medium and high adherence groups respectively (*p* < 0.05).

### Adherence as measured by the MMAS-8 ordinal scores

**Fig. 4:**
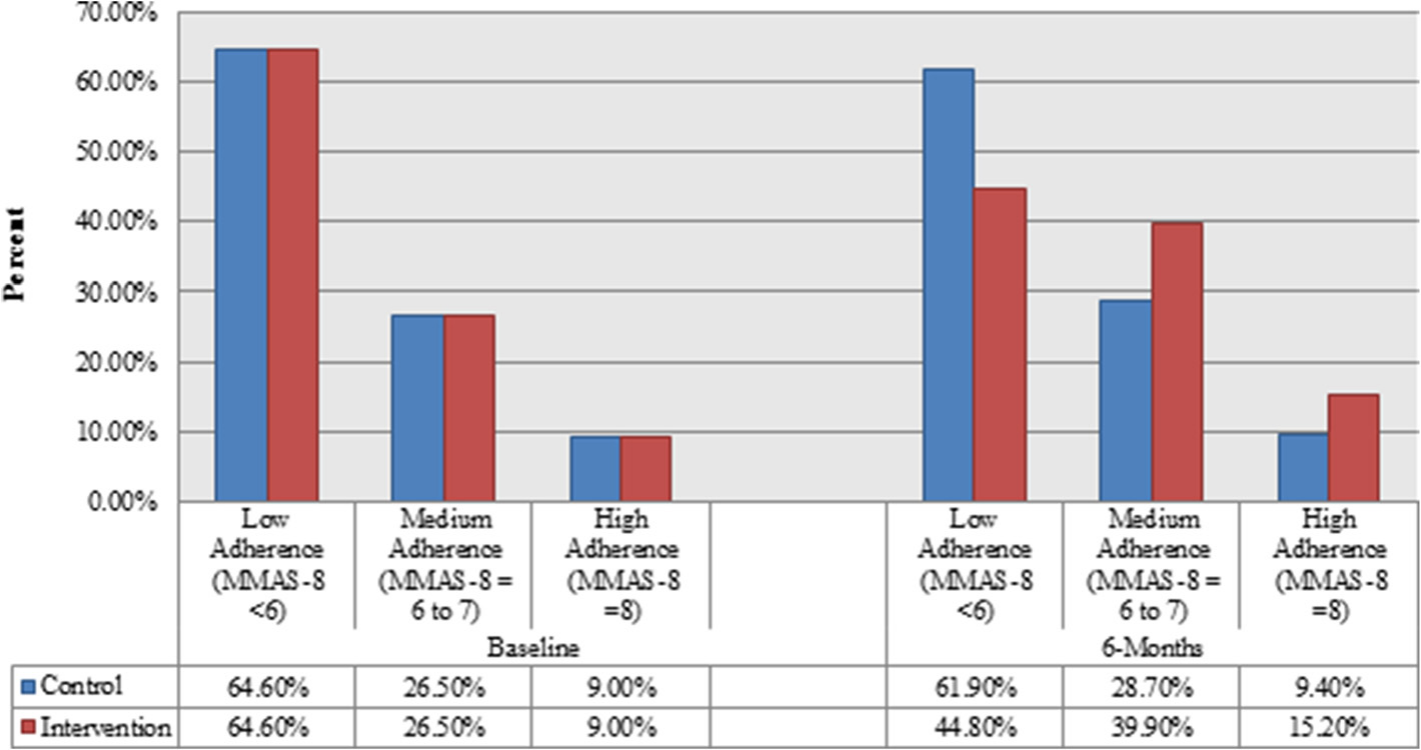
MMAS-8 adherence levels at baseline and 6-months

Descriptive statistics of the data analysed showed a significant increase in the adherence levels in the interventional arm. The percentage of patients scoring low adherence MMAS-8 scores in the interventional group dropped from 64.60 % at baseline to 44.80 % at 6-months (*p* = 0.01). Meanwhile there was no obvious change in the adherence scores at baseline and at 6-months in the control group (Fig. 4).

### Interventional arm

Based on the study data, the Wilcoxon signed-rank test showed that at 6 months, the educational 30-min session on diabetes and adherence to medication did elicit a statistically significant change in adherence levels in patients enrolled in the intervention arm (*Z* = −6.187, *p* <0.001). The median MMAS-8 Score rating was 1.0 (Low adherence) pre-intervention and 2.0 (medium adherence) post-intervention.

### Control arm

On the other hand, the Wilcoxon signed-rank test showed that at 6 months, there was no statistically significant change in adherence levels in patients in the control group (*Z* = −1.528, *p* =0.127). The median MMAS-8 Score rating was 1.0 (Low adherence) at baseline and at 6 months.

### Changes in HbA1c

The mean HbA1c at 6 months was compared to the baseline mean HbA1c in both the Intervention and Control groups using the paired samples T-test. The T-test showed a significant decrease in mean HbA1c at 6-months in the intervention group from baseline (8.5 vs 7.2 % *p* < 0.001). At the same time there was a non-significant decrease in HbA1cn the control group (8.50 vs 8.45 % *p* = 0.079) Table 4.

**Table 4:** Mean HbA1c levels at baseline and 6-months

		Mean	N	Std. Deviation	Std. Error Mean
Control	Baseline HbA1c(%)	8.5018	223	1.39820	.09363
	6-months HbA1c(%)	8.4515	223	1.35603	.09081
Intervention	Baseline HbA1c(%)	8.4892	223	1.42859	.09567
	6-months HbA1c(%)	7.1533	223	1.63739	.10965

## Discussion

Limited data exist on the adherence of diabetics in the United Arab Emirates to their medication prescribed by their doctors as well as on the factors influencing their adherence. This single centre case control study of 442 patients in the United Arab Emirates primarily looked at the utility of an educational session at increasing the levels of medication adherence among patients with diabetes. The results showed an increase in the MMAS-8 adherence scores with the percentage of non-adherents dropping from 65 to 45 %.

Studies in other countries have demonstrated poor adherence rates of medication among diabetics and patients suffering with other chronic conditions. Al Mazroui demonstrated a significant reduction in the levels of HbA1c among diabetics receiving an intensive educational program over a 12 month period of time (baseline vs. 12 months; 95 % confidence interval) of HbA1c8.5 % (8.3, 8.7) vs. 6.9 % (6.7, 7.1) [16]. Reed demonstrated the important role of chronic diabetes clinics in the UAE at improving diabetes outcomes as measured by HbA1c levels and blood pressure. However, neither of the former ‘UAE based’ studies had been designed to study the levels of adherence to medication among diabetics [17].

Despite this lack of data from the United Arab Emirates and the Arab world as a whole, there have been numerous studies from around the world looking at the impact of medication adherence on outcome in patients of chronic medical diseases including diabetes. An American observational study concluded that high adherence levels to medication among diabetics were associated with an overall reduction in healthcare costs [18]. Another retrospective study of over 11,000 patients showed that poorly non-adherent diabetics had higher all-cause hospitalization and all-cause mortality compared to adherent diabetics [9]. Adherence to medication in diabetes is therefore of upmost importance, and identifying factors that lead to poorer medication compliance should be identified to guide healthcare policy.

Non-adherence may arise as a consequence of the patient knowingly disregarding their treatment regimen (active non-adherence), or as a consequence of carelessness or forgetfulness, whereby patients occasionally omit their medication from their daily routine or take that medication later than required (passive non-adherence).

The WHO has identified non-adherence as a multifactorial problem caused by the interplay of factors from any of the following 5 areas: 1) the patient, 2) the condition, 3) the type of therapy prescribed, 4) socioeconomic factors, and 5) health system related factors (WHO, 2001). This complex interaction of different factors leading to non-adherence has been studied by many groups around the world. In Italy, Viana et al. published a meta-analysis of RCTs in patients with type-1 diabetes which focused on psychological, telecare and educational interventions to improve treatment compliance. This meta-analsysis has shown that psychological approaches to improve adherence to diabetes care treatment modestly reduced HbA1c in patients with type 1 diabetes; telecare and education interventions however did not change glycemic control [19].

Koenisberg et al. have shown that reviewing patient goals and treatment regimens at regular intervals (e.g. monthly, biannually, or annually) has been shown to help patients to persist in adhering to their treatment plan [20]. Encouraging patients to monitor their own progress is also important in order for long-term adherence to be successful; it is important, however, for patients to develop their own record system to ensure they maximise their chances of adhering to a long-term treatment plan. Flexibility in treatment options is also important as patients are more likely to achieve their goals using treatments they feel more comfortable with.

Education has been identified as a major barrier to adherence to clinical interventions. Patient counselling to improve patient knowledge of the disease and the benefits of both medical intervention and lifestyle changes has been introduced. Coaching is also now available to encourage positive lifestyle choices, empower patients, develop self-sufficiency and assist patients in identifying and overcoming their own barriers to adherence .

In our cohort of patients the strongest independent predictor of adherence was the patient’s education level. A technical diploma certificate as compared to a primary school level of education was the strongest predictor of adherence (*OR* = 66.1 CI: 6.93 to 630.43); *p* < 0.001). Educational websites have been set up in order to raise health literacy and allow an anonymous forum in which patients can clarify any issues which may be preventing medication adherence. However many patients lack access to such resources. Furthermore, tailoring specific exercise plans and personalising diet plans may also help patients achieve their lifestyle-change goals. This study of type 2 diabetes patients in the United Arab Emirates explored the utility of an educational session on improving medication adherence. The adherence rates among adults with diabetes in this group of patients followed through a primary care setting were extremly low (64.6 % of patients were considered non-adherent with a MMAS-8© adherence score < 6).

The data analysed showed an obvious increase in the adherence levels in the interventional arm. The percentage of patients scoring low adherence MMAS-8 scores in the interventional group dropped from 64.60 % at baseline to 44.80 % at 6-months (Pearson Chi-square *p* = 0.01). Meanwhile there was no obvious change in the adherence scores at baseline and at 6-months in the control group.

Based on the study data, the Wilcoxon signed-rank test showed that at 6 months, the educational 30-min session on diabetes and adherence to medication did elicit a statistically significant change in adherence levels in diabetics enrolled in the intervention arm (*Z* = −6.187, *p* <0.001). The median MMAS-8 Score rating was 1.0 (Low adherence) pre-intervention and 2.0 (medium adherence) post-intervention.

The patients in this study were individually subjected to the educational session which was both time-consuming and man-power intensive. This could be facilitated in the future by asking patients to attend group sessions that could also touch upon other aspects of diabetes besides medication compliance. The Desmond trial showed that a structured group education programme for patients with newly diagnosed type 2 diabetes resulted in greater improvements in weight loss and smoking cessation and positive improvements in beliefs about illness but there were no differences in HA1c levels up to 12 months after diagnosis [21]. Trento et al. has also shown in an RCT over a 5-year period that group care and education of patients with diabetes was associated with better quality of life and knowledge of diabetes compared to control individual care [22].

The intial interview consisted of only 30 min of “one-on-one” time between the interviewer and the patient. This time is shorter when compared to the time utilitized in group intervention programmes. For example, Weinger et al. has shown that a structured behavioral intervention consisting of five 2-hour sessions, delivered over 6 weeks, of highly structured behavior-based activities and information was effective in improving glycemia in adults with long-duration diabetes. The limitations of this study include the small size, which despite meeting the pre-determined study size sample predicted before starting the study would continue to be a source of population bias error. The MMAS is a popular, easy and economical method of data collection, facilitating the collection of a large amount of data in a short period of time. Furthermore, the questions are purposely phrased to avoid the ‘yes-saying’ bias as it is known that patients feel they should provide healthcare providers with a positive response. However, there are limitations to this method of data collection; the MMAS does not account for personal or lifestyle factors (e.g. age, physical ability, means of transport, known methods of communication, etc.), and the outcome of these questions can be biased by patients supplying false information. There was no direct measurement of adherence to medication however the MMAS-8 score has been well validated in measuring medication adherence in diabetes and other chronic conditions.

## Conclusion

Adherence to medication among diabetics in the U.A.E. continues to be low. In this study there was a significant improvement in the adherence to medication in diabetics taking part in an educational session focusing on the importance of anti-diabetes medication and adherence to it. The results of such a pilot program would be of upmost value in supporting the implantation of bigger trials or even a national educational program in the UAE which up to today is still lacking. Public health policy makers in the U.A.E. should be equipped and ready with all the knowledge to get services ramped up quickly as the ‘Diabetes Tsunami’ hits its shores.

## Abbreviations

DASS: Depression, anxiety and stress scale
HbA1c: Glycated haemoglobin alpha concentration
IPAQ: International physical activity questionnaire
MMAS-8: Morisky medication adherence scale- 8
U.A.E.: United Arab Emirates
WHO: World health organization
